# Supplementary material for: Identification of Key Prognostic Genes of Triple Negative Breast Cancer by LASSO-Based Machine Learning and Bioinformatics Analysis
Source: Genes (Basel). 2022 May 18;13(5):902. doi: 10.3390/genes13050902 (PMC9140789; doi:10.3390/genes13050902)
Supplement: Supplementary file 1 [file genes-13-00902-s001.zip › genes-1673609-supplementary.pdf]

**Supplementary Table S1.** The demographic information of the 229 samples.

| Total (n=229) | TNBC (n=116) | Normal (n=113) | *p*-value |
|---|---|---|---|
| | n(%) | n(%) | |
| **Age,years** | | | 0.1151 |
| <51 | 46 (39.7) | 41 (36.3) | |
| 51-70 | 57 (49.1) | 50 (44.2) | |
| 70< | 13 (11.2) | 22 (19.5) | |
| **Race** | | | |
| Asia | 8 (7) | 1 (1.0) | |
| Black or african american | 32 (27.6) | 6 (5.0) | |
| White | 69 (59.4) | 105 (93.0) | |
| Not reported | 7 (6) | 1 (1.0) | |
| **Pathologic stage** | | | |
| StageI | 19 (16.4) | | |
| StageII | 73 (63.9) | | |
| Stage III | 19 (16.4) | | |
| Stage IV | 2 (1.7) | | |
| Not reported | 3 (2.5) | | |
| **Metastasis** | | | |
| M0 | 99 (85.3) | | |
| M1 | 2 (1.7) | | |
| Mx | 15 (13.0) | | |
| **Lymph Node** | | | |
| N0 | 74 (63.8) | | |
| N1 | 26 (22.4) | | |
| N2 | 12 (10.3) | | |
| N3 | 4 (3.5) | | |
| **T stage** | | | |
| T1 | 26 (22.4) | | |
| T2 | 74 (63.8) | | |
| T3 | 12 (12.3) | | |
| T4 | 4 (3.5) | | |
